# Optimization of Enzyme-Assisted Extraction and Purification of Flavonoids from *Pinus koraiensis* Nut-Coated Film and Antioxidant Activity Evaluation

**DOI:** 10.3390/molecules26071950

**Published:** 2021-03-30

**Authors:** Mingyan Zhang, Wuchao Ma, Chao Wang, Ximing Yang, Yuhang Lou, Xinxiu Xia, Hongyan Xu

**Affiliations:** 1 Department of Food Science and Engineering, Agricultural College, Yanbian University, Yanji 133000, China; 15981394086@163.com (M.Z.); 2019010605@ybu.edu.cn (X.Y.); louyuhang0213@163.com (Y.L.); x17853516037@163.com (X.X.); 2 Department of Food Science and Engineering, Fusion College, Yanbian University, Yanji 133000, China; wangchao20210228@163.com; 3 Department of Food Science and Technology, Food College, Dalian Polytechnic University, Dalian 116000, China; mawuchao66@163.com

**Keywords:** *Pinus koraiensis* nut-coated film, flavonoids, enzyme-assisted extraction, response surface method, macroporous resin, antioxidant activity

## Abstract

*Pinus koraiensis* nut-coated film is a kind of by-product of nut processing, which has been shown to contain flavonoids, polyphenols, and other substances that can be used to produce natural antioxidant extracts. In this study, response surface methodology (RSM) was used to optimize the extraction process of flavonoids of *P. koraiensis* nut-coated film (PNF), and macroporous resin HPD600 was used to purify PNF (P-PNF). Its antioxidant activity was examined by DPPH (1,1-diphenyl-2-picrylhydrazyl) radical scavenging capacity, oxygen free radical absorption capacity (ORAC), total oxygen radical capture (TRAP), and iron ion reduction capacity. Under the ideal extraction conditions comprising a cellulase dosage of 90 U/g, a material/liquid ratio of 1:20 (g/mL), and an extraction time of 2 h, the PNF yield was 3.37%. Purification conditions were sample concentration of 2.0 mg/mL, pH of 5, water washing volume of 3 bed volume (BV), eluent ethanol concentration of 50%, and volume of 2 BV. The P-PNF recovery was 84.32%, and purity increased from 33.80% to 61.70%. Additionally, P-PNF showed increased antioxidant activity compared to PNF. Cumulatively, this study obtained the optimal values for the process parameters in order to achieve the maximum rates of extraction of PNF for economically optimal production at an industrial scale.

## 1. Introduction

*Pinus koraiensis*, the main tree species found on Changbai Mountain in northeastern China, is an important source of wood and a traditional medicinal plant. *P. koraiensis* nut, the seed nut of *P. koraiensis*, is a kind of nutritional and delicious food, usually used in pastry and traditional dishes, and as a snack food [1]. It is rich in lipids, protein, essential amino acids, polyphenols, and other substances [2]. Among the nutrients, *P. koraiensis* nut contains high oil content with unsaturated fatty acid up to 85% [3]. In particular, it was reported that the *P. koraiensis* nut oil contains pinolenic acid, which has been shown to have good bioactivity and could reduce the effects of high blood pressure [4]. Additionally, the protein [5] and polysaccharide [6] components of *P. koraiensis* nut were also proved to have good antioxidant and anti-inflammatory activities. Based on these beneficial ingredients, the deep processing industry of *P. koraiensis* nut has developed rapidly in recent years. What is noteworthy is that in the processing of *P. koraiensis* nut, a large number of by-products are produced, such as the *P. koraiensis* nut-coated film. However, currently, few studies have actually investigated the value of this nut-coated film. In the meantime, the nut-coated film continues to be discarded, potentially wasting a valuable resource. Our previous research found that the *P. koraiensis* nut-coated film contained polyphenols, flavonoids, and other ingredients (Table A1 in Appendix A), having the research value. Flavonoids, a class of plant-derived dietary polyphenols, are consumed in the human diet in fruits, cereals, spices, and other plant-based products [7,8]. Flavonoids have a broad range of pharmacological properties, including anti-inflammatory, antitumor [9], stabilization of immune cells [10], and others, which are associated with the antioxidant activity of flavonoids [11]. Hence, designing a method for the effective extraction of flavonoids from *P. koraiensis* nut-coated film (PNF) will improve the resource utilization rate.

Commonly employed extraction methods for flavonoids include solvent extraction, ultrasonic assisted extraction, microwave assisted extraction, enzyme-assisted extraction, and others [12]. Enzyme-assisted extraction offers advantages such as high efficiency and mild conditions, while ensuring maintenance of the properties and stability of the extraction [13]. Hence, it has been widely used in the extraction of biologically active ingredients from plants. Moreover, the activity of flavonoids is reportedly positively associated with its purity [14], highlighting the need for an appropriate purification method for PNF following their extraction. Macroporous resin, an organic polymer adsorbent, is one such method that is commonly used for purifying flavonoids, which offers the advantages of a simple protocol and good physicochemical stability [15].

The current study sought to verify the feasibility of enzyme-assisted extraction and macroporous resin purification for PNF, while determining the ideal extraction conditions (cellulase dosage, material/liquid ratio, and extraction time) and purification conditions (sample concentration, pH, eluent concentration, and washing volume). To this end, the in vitro antioxidant activities of flavonoids from the *P. koraiensis* nut-coated film before and after purification were assessed on the basis of DPPH radical scavenging capacity, total oxygen radical capture (TRAP) value, oxygen free radical absorption capacity (ORAC) value, and iron ion reduction capacity.

## 2. Results

### 2.1. Optimization of the Extraction Conditions

The following test schemes and results were obtained by a three-factor analysis of cellulase dosage, material/liquid ratio, and extraction time by the response surface method (RSM; Table 1).

The regression model obtained by regression fitting the result of PNF yield was:

Y = 3.41667 + 0.00875X_1_ + 0.16000X_2_ − 0.02375X_3_ + 0.04042X_1_^2^ − 0.29208X_2_^2^ − 0.18458X_3_^2^ + 0.01000X_1_X_2_ − 0.00250X_1_X_3_ + 0.01500X_2_X_3_ (1)

Using the same method, the formula determined for ABTS^+^ clearance rate was:

Y = 87.6400 − 0.2737X_1_ + 2.4438X_2_ − 0.7125X_3_ − 3.1463X_1_^2^ − 5.2263X_2_^2^ + 0.3612X_3_^2^ + 1.8225X_1_X_2_ − 0.2550X_1_X_3_ + 0.2200X_2_X_3_ (2)

*R*^2^ (coefficient of determination), an indicator used as a measure of model suitability and model, can indicate the relationship between the experimental and predicted values of the response. The closer *R*^2^ is to 1, the more accurate the model is [16]. The *R*^2^ of the regression model were 89.63% and 97.78%, respectively. The *p*-values of the regression model were all significant (*p* < 0.05), while the lack-of-fit were not significant (*p* > 0.05) (Table 2). The quadratic model best described the true relationship between each factor and the response value to fit the experimental results.

Table 3 presents the results of a comprehensive analysis for the response surface, highlighting the effects of various factors on PNF yield in the following order: material/liquid ratio > extraction time > dosage of cellulase. The results of X_2_, X_2_^2^, and X_3_^2^ for PNF yield were significant, while the other terms were not. The interaction terms were X_2_X_3_, X_1_X_2_, X_1_X_3_ in order of influence, and the effects were not significant, indicating that the factors were not linear. The results of X_2_, X_1_^2^, and X_2_^2^ for ABTS^+^ clearance rate were significant, while the other terms were not. The influence of X_2_ was significant, as was the influence of the squared items X_1_^2^ and X_2_^2^. The interaction items were arranged in the order X_1_X_2_, X_1_X_3_, and X_2_X_3_, with X_1_X_2_ found to have a significant influence.

**Figure 1:**
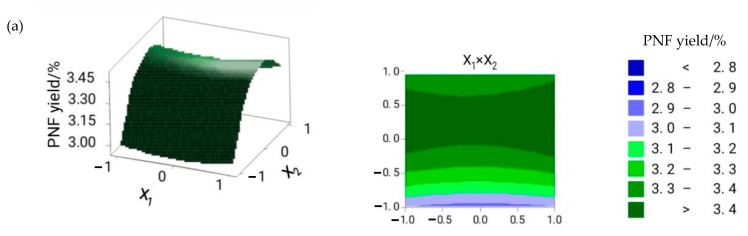

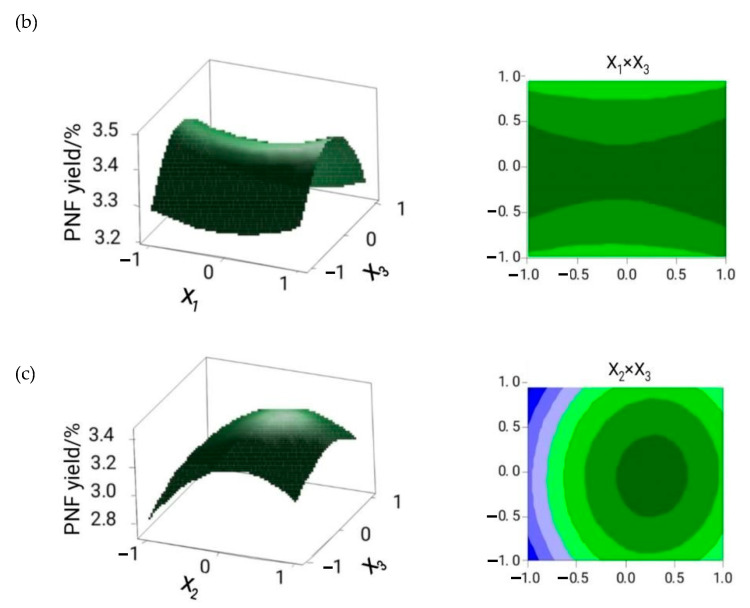
The effect of cellulase dosage (X_1_), material/liquid ratio (X_2_), and extraction time (X_3_) on flavonoid yield. (**a**) The interaction between cellulase dosage and material/liquid ratio; (**b**) the interaction between cellulase dosage and extraction time; (**c**) the interaction between material/liquid ratio and extraction time.

**Figure 2:**
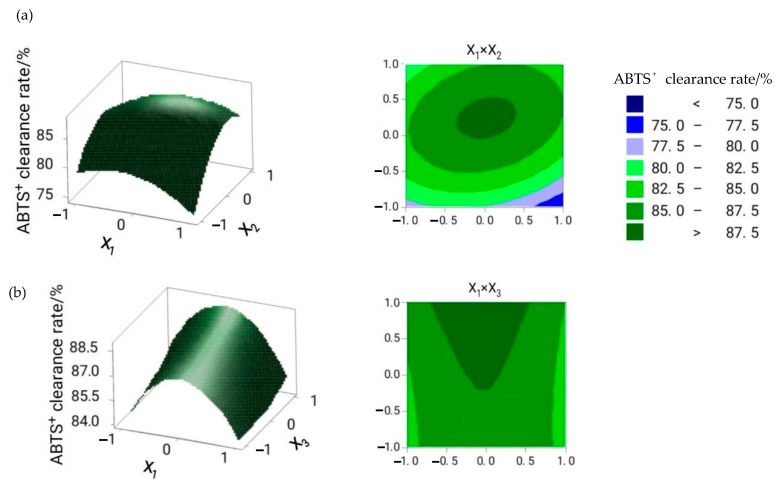

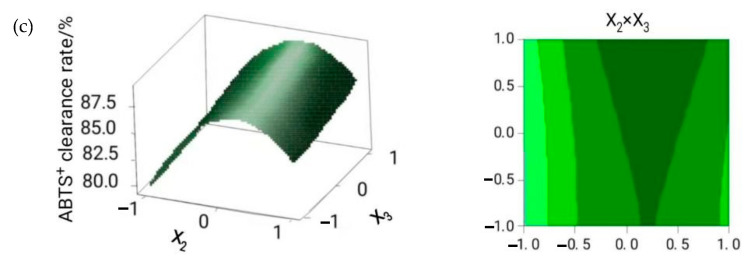
The effect of cellulase dosage (X_1_), material/liquid ratio (X_2_), and extraction time (X_3_) on ABTS^+^ clearance rate. (**a**) The interaction between cellulase dosage and material/liquid ratio; (**b**) the interaction between cellulase dosage and extraction time; (**c**) the interaction between material/liquid ratio and extraction time.

The three-dimensional response surface and the two-dimensional contour plots were provided as graphical representations of the regression equation [17]. Figure 1 shows the influence on flavonoid yield. When the extraction time was 2 h, and the material/liquid ratio was gradually increasing, the flavonoid yield first increased and then decreased, while no significant effect on cellulase dosage was observed (Figure 1a). When the material/liquid ratio was 1:20, with a gradual increase in extraction time, the flavonoid yield first increased and subsequently decreased, while no significant impact on enzyme dosage was observed (Figure 1b). Figure 1c presents the effects of the material/liquid ratio, extraction time, and their reciprocal interaction on the flavonoid yield at a cellulase dosage of 90 U/g. As the material/liquid ratio and extraction time increased, the flavonoid yield initially increased and then decreased. The interaction of these two factors was better than that of X_1_X_2_ and X_1_X_3_. Figure 2 depicts the effect of these three factors on ABTS^+^ clearance rate. Results showed that the material/liquid ratio and extraction time significantly influenced the ABTS^+^ clearance rate (Figure 2a).

According to the regression model, the optimal process parameters were determined as follows: cellulase dosage was 90.13 U/g, material/liquid ratio was 1:21.6 (g/mL), extraction time was 1.98 h, acetone volume fraction was 50%, extraction temperature was 55 ℃, and pH was 4.5. The theoretical value of the PNF yield predicted by the regression model was 3.44%, and the ABTS^+^ clearance rate was 87.36%. Taking practical feasibility into account, the extraction conditions of PNF were adjusted to a theoretical value of cellulase dosage of 90 U/g, a material/liquid ratio of 1:22, extraction time of 2 h, extraction temperature of 55 °C, acetone volume fraction of 50%, and pH of 4.5. Five parallel experiments were performed, and the average yield of total flavonoids was 3.37% with an RSD value of 1.78%. The average ABTS^+^ clearance rate value was 88.14% with an RSD value of 1.70%. The verification results were similar to the predicted values, indicating that the model was well fitted to the experiment, and the extraction conditions of PNF obtained by the RSM were reliable.

### 2.2. Optimization of the Purification Conditions

**Figure A1:**
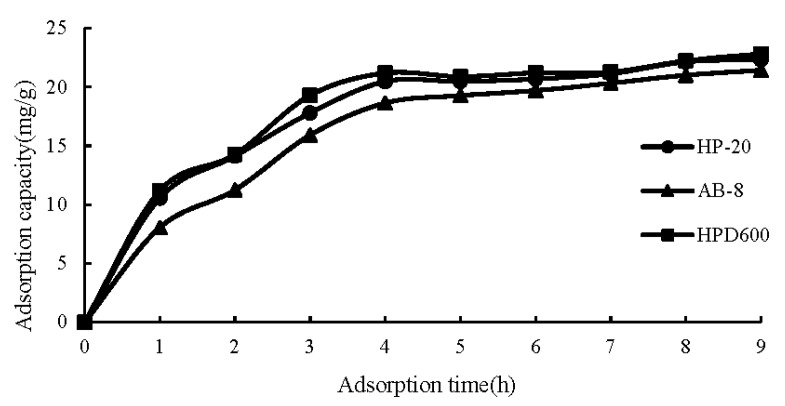
Static adsorption kinetic curves.

**Figure A2:**
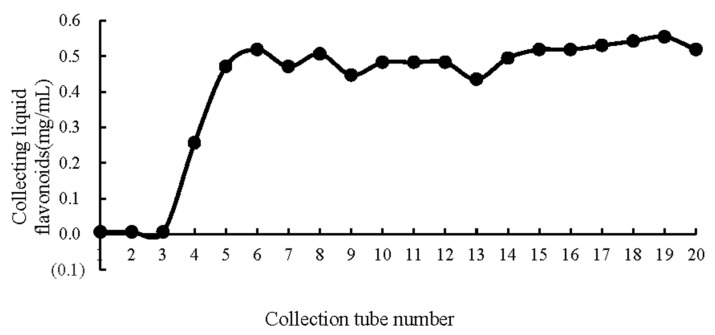
Dynamic adsorption kinetic curves of HPD600.

**Figure A3:**
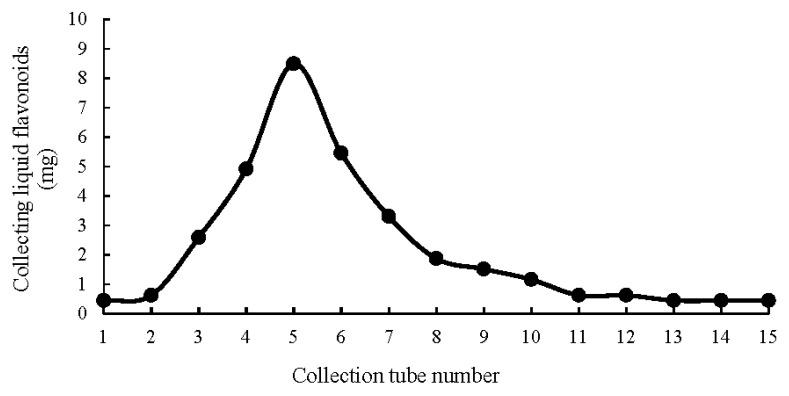
Curves of dynamic desorption.

PNF was treated with macroporous resin to obtain purified flavonoids (P-PNF). Three macroporous resins selected after screening were all fast-balanced, and the adsorption equilibrium was reached after 4 h (Figure A1), resulting in the adsorption time being defined as 4 h. Since HPD600 had the highest adsorption rate, it was selected for PNF purification, and the adsorption time was determined to be 4 h. The extract solution (4.91 mg/mL) was loaded into the column at a flow rate of 1.0 mL/min. From the start of the assay, the effluent was collected at intervals of 5 mL, and the concentration was measured to prepare dynamic adsorption kinetics for P-PNF from HPD600 (Figure A2 in Appendix A). No flavonoids were present in the first three collection tubes; however, they were detected in the fourth collection tube, indicating that the flavonoids began to flow. The mass concentration of flavonoids was found to be 0.52 mg/mL in the sixth collection tube, after which their concentration of flavonoids remained relatively unchanged in the subsequent collection tubes, indicating that the adsorption was substantially saturated. To ensure that there was no waste due to leakage, and to take full advantage of the PNF and macroporous resin, 15 mL was selected as the maximum loading. PNF with a pH of 6 and a concentration of 1.5 mg/mL was dynamically loaded, and the flow rate was controlled at 1 mL/min. Then, macroporous resin was washed with 4 bed volume (BV) distilled water followed by 90% ethanol solution. The eluent was collected in one portion per 10 mL for a total of 15 portions (Figure A3 in Appendix A). As the eluent volume increased, the flavonoids in the collection tube gradually increased. In the fifth tube when the liquid volume reached approximately 1 BV, the flavonoid content was the highest. Thereafter, the flavonoid content in the eluate rapidly declined. Ultimately, an eluent amount of 2 BV was determined to fully elute the flavonoid on the macroporous resin and was therefore set as the optimum amount of eluent.

According to the results of the single factor test, sample concentration, pH, eluent concentration, and water washing volume were selected as factors. The L_9_ (3^4^) orthogonal table was designed and analyzed by variance. The impact of various factors on flavonoid recovery were as follows: sample concentration > pH > eluent concentration > water washing volume. Among them, the eluent concentration and pH had extremely significant (*p* < 0.01) effects on purified flavonoids (Table 4 and Table 5). The optimal condition for flavonoid purification by macroporous resin was A_3_B_1_C_2_D_1_, i.e., the sample concentration of 2.0 mg/mL, pH of 5, ethanol eluent concentration of 50%, and water washing volume of 3 BV. Accordingly, the verification experiment was carried out under the above experimental conditions. The parallel experiment was averaged three times to obtain a flavonoid recovery rate of 84.32%, which was greater than the results in the orthogonal experiment. The purity was increased from 33.80% of PNF to 61.70% of P-PNF.

### 2.3. In Vitro Antioxidant Activity

**Figure 3:**
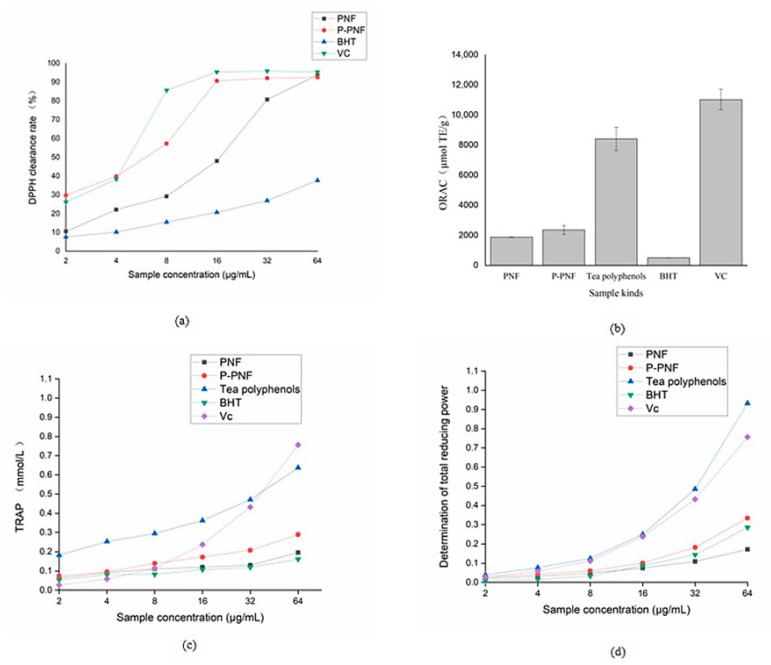
In vitro antioxidant activity of *P. koraiensis* nut-coated film (PNF) and purified P-PNF. (**a**) DPPH radical scavenging activity; (**b**) oxygen free radical absorption capacity (ORAC) value; (**c**) total oxygen radical capture (TRAP) value; (**d**) iron ion reduction capacity.

The DPPH radical scavenging activity in each sample is shown in Figure 3a, while ascorbic acid (VC) and butylated hydroxytoluene (BHT) served as the positive controls. The IC_50_ values of PNF, P-PNF, BHT, and VC of DPPH free radical clearance activity were 9.832, 8.636, 174.911, and 5.502 µg/mL, respectively. A dose-dependent relationship was observed in the DPPH scavenging activity of PNF and P-PNF, that is, an increase in concentration was synonymous with an increase in scavenging capacity. When the concentration ranged from 2 to 32 µg/mL, the scavenging ability of P-PNF was consistently superior to that of PNF and below the VC. However, when the concentration was greater than 16 µg/mL, the free radical scavenging ability of VC and P-PNF on DPPH tended to balance. In particular, when the concentration reached 64 μg/mL, the antioxidant effect of PNF, P-PNF, and VC was almost the same, which is because the DPPH free radical scavenging ability of these substances reached saturation.

The standard Trolox curve equation was *Y* = 0.64390*X* − 0.50745, *R*^2^ = 0.99369. Figure 3b shows that the ORAC value of VC (11,029 mol TE/g) was the highest, followed by that of tea polyphenols (8,412 mol TE/g) and P-PNF (2,357 mol TE/g). Compared with the ORAC value of PNF (1,741 mol TE/g), the ORAC of P-PNF was significantly higher (*p* < 0.05), indicating that purification could effectively improve the ORAC value of PNF. The TRAP value is the reaction between the free radicals generated by AAPH and ABTS interference by the antioxidant. The TRAP of every sample was determined, and the results are shown in Figure 3c. The TRAP value of each sample increased with increasing mass concentration and showed a dose-dependent relationship. Moreover, the TRAP value of P-PNF was higher than that of BHT, indicating that P-PNF had better antioxidant capacity.

The iron ion reduction capacity is an important index for determining the antioxidant capacity of plants. This method of analysis is fast and effective and thus widely used. Figure 3d shows a dose-dependent relationship between sample iron ion reduction capacity and sample concentration. P-PNF showed good iron ion reduction capacity (0.029 ± 4.88%–0.334 ± 0.63%), which was stronger than that of PNF (0.021 ± 6.73%–0.172 ± 2.06%) and BHT (0.008 ± 3.53%–0.285 ± 0.49%) at the same concentration; however, it was slightly lower than VC (0.027 ± 5.24%–0.756 ± 0.19%), indicating the P-PNF had strong reducing power.

## 3. Discussion

In this study, the extraction and purification conditions of PNF were optimized, and the antioxidant activity of flavonoids before and after purification were detected.

Extraction is a key step in the separation of bioactive compounds from plant materials. A proper selection of conditions, including an appropriate technology and solvents, can yield highly reactive products. Currently, there are many methods for the preparation of flavonoids, including water extraction, solvent extraction, supercritical fluid extraction, ultrasonic extraction, microwave-assisted extraction, etc. [18]. The advantages of these extraction methods include increased extraction yield and decreased extraction time. However, the disadvantages of these are obvious, including expensive equipment, small scale, and environmental pollution, which are not suitable for the application of by-product processing [19]. Consequently, with the pursuit of the concept of “green chemistry”, people are looking for environmentally friendly and efficient extraction methods to improve the recovery rate and biological utilization. Enzymatic extraction has shown several advantages because of its environmental friendliness and enhanced extraction ability or recovery of targeted compounds under mild processing conditions [20]. Enzyme reaction can effectively destroy cellulose, hemicellulose, pectin, and other material structures of plant cells, so that the flavonoids are easier to be released and the extraction rate is increased [21]. Cellulase and pectinase are two kinds of biological enzymes that are widely used at present. Studies have shown that under the same conditions, the extraction yield of *Zizania latifolia* flavonoid with an enzyme mixture (cellulose, hemicellulose, and pectinase) treatment (17.5%) was the highest and resulted in a significant increase as compared to the non-hydrolyzed extract (10.7%) [22]. Cellulase-assisted acetone extraction was used to extract PNF in our study, and the yield reached 3.37%. In addition, the ABTS free radical scavenging rate was tested in order to verify the antioxidant activity of PNF. The results showed that the ABTS free radical scavenging rate of PNF was 88.14%, which strongly proved that PNF had the potential to be a natural antioxidant.

Natural antioxidants are widely used as food additives for anti-corrosion, sterilization, the blocking of oxidation reaction, etc., so as to extend the shelf life of food [23]. The by-products (such as leaves, branches, cones, shells, etc.) from the processing of forest resources are rich reservoirs of plant antioxidants, which are worthwhile to study and utilize [24]. Flavonoids, as good natural antioxidants, can eliminate nitrogen and reactive oxygen species by scavenging free radicals [25], which is related to their structural conformations, p-electron delocalization, potential polarizability, hydroxyl groups distributed in flexible ring B, and functional groups [26]. In addition, the purified flavonoids have stronger antioxidant effects than the crude extracts, which is related to the purity of flavonoids. After purification by macroporous resin, the antioxidant capacity of flavonoids from *Glycyrrhiza glabra* L. leaf was increased by 2–3 times [27]. The scavenging rates of DPPH and ABTS free radicals of 100 μg/mL purified flavonoids from *Moringa oleifera* leaves were 85.99% and 84.79%, respectively [28]. Thus, in order to further study the activity of the extracts, the components needed to be purified and enriched before processing. There are several methods for the enrichment of active constituents, such as membrane filtration [29], ion exchange [30], expanded bed adsorption [31], and resin adsorption [32]. Comparatively, macroporous resin adsorption seems to be the most suitable method due to its high efficiency, environmental protection, low cost, and so on [33], and is suitable for industrial and large-scale production. The purity of flavonoids from *Scutellariae barbatae* herbs was increased by 1.16 times by macroporous resin [34], and after purification with macroporous resin, the purity of oak cup flavonoids reached 77.00% [35]. These studies indicated that macroporous resin can effectively enrich and purify flavonoids from raw materials. HPD600 macroporous resin was selected to purify PNF in our study, and the purity was increased from 33.80% to 61.70%. The results of antioxidant testing showed that both PNF and P-PNF had good antioxidant activity, and the antioxidant activity of P-PNF was much better than that of PNF. Additionally, in general, the antioxidant effect of P-PNF was better than that of BHT (Figure 3). BHT, as one of the widely used synthetic antioxidants, despite the low cost advantage, is considered to have a cancer risk [36]. Consequently, as a natural antioxidant obtained from processing by-products, P-PNF has wide application prospects. Metal ions and water have important mechanisms in the process of radical scavenging capacity [37]. Flavonoids can form complexes with Fe and Cu and thus have a stronger reaction with DPPH when compared to compounds without metal ions [38]. In the presence of water, the kinetics of the DPPH radical reaction increases. The water may be related to the impurities in the extraction solvent or the humidity of the extracted plant material [39]. ORAC value and TRAP are the rapid methods to determine total antioxidant capacity in biological samples [40]. The iron ion reduction capacity is generally associated with the presence of antioxidant agents, which exert their effect by breaking the free radical chains via hydrogen atom donation. In terms of concentration, there was a dose-dependent relationship between the concentration and the antioxidant activity of P-PNF. From a structural point of view, the antioxidant activity of flavonoids depends on the number and position of substituents in the molecule [41]. Therefore, in order to further explain the antioxidant activity of P-PNF, it is necessary to conduct subsequent tests to analyze the structure of P-PNF. In addition, the cell or animal tests are also considered to arrange to verify the antioxidant activity in vivo so as to improve the utilization value of *P. koraiensis* nut-coated film.

## 4. Materials and Methods

### 4.1. Plant Materials

*P. koraiensis* nut was provided by Meihekou Siquan Native Products Co., Ltd. (Jilin, China). The sample was dried at 50 °C using a 1400032S draught drying cabinet (Shanghai Hengke Instrument Co., Ltd., Shanghai, China), ground by an SB-10A multifunctional grinder (Shanghai Puheng Information Technology Co., Ltd., Shanghai, China), and passed through a 40 mesh screen to extract the flavonoids.

### 4.2. Extraction Conditions Optimization

#### 4.2.1. Enzyme-Assisted Extraction

PNF was extracted by enzyme-assisted solvent extraction. Acetone (analytically pure, Tianjin Kemiou Chemical Reagent Co., Ltd., Tianjin, China) was chosen as the extraction solvent and cellulase (30 U/mg, from *Trichoderma viride*) (Beijing Boao Tuoda Science and Technology Co., Ltd., Beijing, China) as the hydrolase, and 0.1 mol/L HCl solution was used to adjust pH. Cellulase and acetone were added to the sample and left to soak at 25 ℃ for 0.5 h. PNF was obtained by filtration, collection of the filtrate, enzyme deactivation at 90 ℃ for 10 min, and subsequently freeze drying (LGJ-10 freezer dryer; Beijing Sihuan Scientific Instrument Factory, Beijing, China). Rutin standard sample was purchased from the China National Institute for the Control of Pharmaceutical and Biological Products and NaNO_2_-Al(NO_3_)_3_ colorimetry was used to construct the standard curve [42]. The linear equation was *Y* = 0.014*X* + 0.0167, *R*^2^ = 0.9958. A constant solution volume of 2 mL was used to calculate the concentration of flavonoids in the sample solution according to the linear equation. The flavonoid yield was calculated as follows:

(3) $$\mathrm{Flavonoids}\;\mathrm{yield}\;\%\;=\;\frac{\mathrm{CNV}}{m}\;\times \;100\%$$

where V is the volume of the sample solution (mL), N is the determined dilution, C is flavonoid concentration calculated according to the standard curve, and m is mass of the sample (g).

#### 4.2.2. RSM Experimental Design

The optimum extraction conditions for PNF were optimized by RSM. A single factor test was performed using six factors including cellulase dosage (60–130 U/g), acetone volume fraction (10–80%), material/liquid ratio (1:10–1:70, g/mL), extraction time (1–4 h), extraction temperature (30–60 °C), and pH (3.5–6.5). Following the analysis and comparison of the single factor test results, three factors were selected as independent variables: cellulase dosage (X_1_), material/liquid ratio (X_2_), and extraction time (X_3_). Each factor was assessed at three levels.

#### 4.2.3. ABTS Radical Scavenging Assay

The ABTS^+^ clearance rate of PNF was measured according to the method of Pan [43] with some minor modifications. ABTS (2,2′-azino-bis(3-ethylbenzothiazoline-6-sulfonicacid); 7.4 mmol/L) (Beijing Boao Tuoda Science and Technology Co., Ltd., Beijing, China) and potassium persulphate (2.6 mmol/L; Tianjin Fuchen Chemical Reagent Factory, Tianjin, China) were mixed at a ratio of 1:1 (*v*:*v*). The mixture was prepared into ABTS reserve solution at room temperature in the dark for 24 h. ABTS working solution was obtained by diluting ABTS reserve solution with 60% ethanol (analytically pure, Tianjin Kemiou Chemical Reagent Co., Ltd., Tianjin, China). The ABTS working solution (3.8 mL) was then mixed with 0.1 mL of the sample at room temperature for 6 min, after which the absorbance was measured at 734 nm and the clearance rate was calculated as follows:

(4) $$\mathrm{Clearance}\;\mathrm{rate}\;\%\;=1\;-\;\frac{A_{1}{\;-\;A}_{2}}{A_{0}}\;\times \;100\%$$

where A_1_ represents the absorbance of the 3.8 mL ABTS and 0.1 mL sample solution mixture, A_2_ is the absorbance of the mixture of 3.8 mL distilled water and 0.1 mL sample solution mixture, and A_0_ is the absorbance of the mixture of 3.8 mL ABTS and 0.1 mL ethanol mixture.

### 4.3. Purification Conditions Optimization

#### 4.3.1. Selection and Preparation of Macroporous Resin

Considering that phenolics contain non-polar phenyl groups and polar multi-hydroxyl groups, five macroporous resins with various polarities were employed, including AB-8, D101 (Tianjin Huida Chemical Co., Ltd., Tianjin, China), HP-20, HPD600, and HPD826 (Beijing Solis Bao Technology Co., Ltd., Beijing, China). The pretreatment of macroporous resins was carried out according to the method of Belwala [44] with minor modifications. All macroporous resins were soaked with 95% ethanol for 24 h before installing the column using the wet method, and 95% ethanol was then used for flow elution on the column. When ethanol effluents were mixed with distilled water without white turbidity, the distilled water was used to wash the ethanol from the column. The resins were then soaked with 2 BV 5% HCl for 3 h and washed with distilled water until neutral. Next, 5% NaOH was used to repeat the procedure in place of HCl. Adsorption and desorption capacities of the five macroporous resins are shown in Figure A1.

The static adsorption kinetics of the three selected kinds of ideal macroporous resins were investigated according to the methods of Jiang [45] with minor modifications. A total of 10 g of each macroporous resin was added to the sample solution (2.75 mg/mL, 100 mL). The mixture was shaken at 25 °C, and 1 mL of the solution was removed each hour to generate the static adsorption kinetic curve.

#### 4.3.2. Purification of PNF

PNF was purified by macroporous resin to obtain P-PNF. Sample concentration (1.0–3.0 mg/mL), pH (4–8), eluent concentration (50–90%), and water washing volume (2–6 BV) were selected as single factor tests. Through the analysis and comparison of the results, the orthogonal experiment for the four factors and three levels was designed to determine the purifying conditions. To achieve this, 1 mL of every 150 mL recovered liquid was taken for absorbance determination. The flavonoid concentration was determined according to the rutin standard curve, and the flavonoid recovery rate was calculated as follows:

(5) $$\mathrm{Flavonoid}\;\mathrm{recovery}\;\%\;=\;\frac{X_{1}V_{1}}{X_{2}V_{2}}\;\times \;100\%$$

where X_1_ is concentration of flavonoids in the recovered liquid (mg/mL), V_1_ is the volume of the recovered liquid (mL), X_2_ is the concentration of flavonoids in the sample liquid (mg/mL), and V_2_ is the sample volume (mL).

P-PNF was dissolved in 60% ethanol, and the concentration of the flavonoids was calculated according to the rutin standard curve, while the purity was calculated as follows:

(6) $$\mathrm{Purity}\;\%\;=\;\frac{\mathrm{CNV}}{m}\;\times \;100\%$$

where C represents the flavonoid mass concentration (mg/mL), N is the dilution factor when the sample liquid is measured, V is the volume of the sample solution (mL), and m is the mass of the sample (mg).

### 4.4. In Vitro Antioxidant Activity

#### 4.4.1. DPPH Radical Scavenging Activity

The method of DPPH radical scavenging activity was measured as suggested by previous reports with appropriate modifications [46]. DPPH (1,1-diphenyl-2-picrylhydrazyl) (Shanghai Yuanye Biotechnology Co., Ltd., Shanghai, China) reserve solution of 0.15 mmol/L was prepared and diluted with anhydrous ethanol to obtain DPPH working solution. A sample solution at a volume of 2 mL was added to 2 mL of DPPH working solution. The mixture was placed in the dark for 30 min and the absorbance was measured by a UV-1800 VIS spectrophotometer (Shanghai Meipuda instrument Co., Ltd., Shanghai, China) at 517 nm. The clearance rate was calculated as follows:

(7) $$\mathrm{Clearance}\;\mathrm{rate}\;\%\;=\left(1\;-\;\frac{A_{1}{\;-\;A}_{2}}{A_{0}}\right)\times \;100\%$$

where A_1_ is the absorbance of the mixture of 2 mL sample solution and 2 mL DPPH, A_2_ is the absorbance of the mixture of 2 mL sample solution and 2 mL ethanol, and A_0_ is the absorbance of the mixture of 2 mL DPPH and 2 mL ethanol.

#### 4.4.2. Determination of ORAC

The ORAC assay was conducted according to the literature with slight modification [47]. First, 79.7 mmol/L AAPH (2,2′-azobis-2-methyl-propanimidamide, dihydrochloride) (Beijing Boao Tuoda Science and Technology Co., Ltd., Beijing, China) solution and 0.0957 mmol/L sodium fluorescein (Beijing Boao Tuoda Science and Technology Co., Ltd., Beijing, China) solution were respectively configured by phosphate buffer (pH 7.4). The phosphate buffer was also used to prepare Trolox (Shanghai Yuanye Biotechnology Co., Ltd., Shanghai, China) solution with different concentrations to determine the standard curve. Then 20 μL sample and 200 μL sodium fluorescein solution were added into each microhole of the 96-well fluorescent plate (Shanghai Jingan Biotechnology Co., Ltd., Shanghai, China) and incubated at 37 °C for 10 min. Finally, 20 μL of AAPH solution was rapidly added to activate the reaction. The Sp-Max 3500FL type multifunctional luciferase marker (Shanghai Shanpu Biological Technology Co., Ltd., Shanghai, China) was used to measure the absorbance. The fluorescence at the excitation wavelength of 485 nm and the emission at 528 nm were monitored once every 270 s for a total of 35 times. A standard curve of fluorescence intensity was recorded as f_1_, f_2_, f_3_..., f_35_. The area under the fluorescence attenuation curve was calculated as follows:

(8) $$\mathrm{AUC}=(0.5\;\times \;\frac{f_{1}}{f_{1}}\;+\;\frac{f_{2}}{f_{1}}\;+\ldots +\;\frac{f_{35}}{f_{1}}\;+\;0.5\;\times \;\frac{f_{35}}{f_{1}})\times \;t$$

where f_1_ is the first fluorescence reading value, f_i_ is intensity multiplied by fluorescence reading value, and t is the time interval (min).

The ORAC value was calculated as follows:

(9) $$\mathrm{ORAC}\;\mathrm{value}\;=\;\frac{{\mathrm{AUC}}_{\mathrm{sample}\;}{-\;\mathrm{AUC}}_{\mathrm{blank}}}{{\mathrm{AUC}}_{\mathrm{Trolox}}{\;-\;\mathrm{AUC}}_{\mathrm{blank}}}\;\times \;\frac{C_{\mathrm{Trolox}}}{C_{\mathrm{sample}}}$$

where AUC_sample_ is the area under the fluorescence attenuation curve of the sample group, AUC_blank_ is the area under the fluorescence attenuation curve of the blank sample group, the area under the fluorescence attenuation curve of AUC_Trolox_ is the Trolox standard antioxidant group, C_Trolox_ is the Trolox concentration (mol/L), and C_Sample_ is the sample concentration (mol/L). Our final ORAC value is represented as moles of TE/g.

#### 4.4.3. Determination of TRAP

TRAP was determined according to the methods described by Shen [48] with slight modification. AAPH and ABTS of a certain mass were respectively weighed and dissolved in the acetate buffer (pH 4.3) and were configured into the AAPH solution and ABTS solution with a concentration of 2 mmol/L and 75 mol/L. Next, 5 mL of the mixed solution was transferred to the test tube followed by 80 μL of the sample solution. The absorbance value at 734 nm wavelength was determined following incubation in a water bath at 25 °C for 15 min. Acetic acid buffer (50 mmol/L) was used as the blank reference, and 1 mmol/L VC (Sinopharm Group Chemical Reagent Co., Ltd., Beijing, China) solution was taken as the standard reference. The antioxidant parameters of TRAP were calculated as follows:

(10) $$\mathrm{TRAP}\;\mathrm{value}\;=\;\frac{A_{\mathrm{sample}}{\;-\;A}_{\mathrm{blank}}}{A_{\mathrm{standard}}{\;-\;A}_{\mathrm{blank}}}{\;\times \;C}_{\mathrm{standard}}$$

where C is VC concentration and A is absorption value. TRAP value is equivalent to the mmol/L of VC.

#### 4.4.4. Determination of Iron Ion Reduction Capacity

Iron ion reduction capacity was assayed according to the methods described by Atki [49] with minor modifications. Briefly, 1.5 mL of sample solution, 2 mL of phosphate buffer (pH 6.6), and 2 mL of 5% potassium ferricyanide (Tianjin Kemiou Chemical Reagent Co., Ltd., Tianjin, China) were mixed. After the mixture was bathed at 50 °C for 20 min, 2.5 mL of 10% trichloroacetic acid (analytically pure, Tianjin Kemiou Chemical Reagent Co., Ltd., Tianjin, China) solution was added and centrifuged for 10 min (4000 rpm). The supernatant (2 mL) was taken and combined with 2 mL of distilled water and 0.3 mL of 0.1% FeCl_3_ (analytically pure, Tianjin Kemiou Chemical Reagent Co., Ltd., Tianjin, China) to develop the color. The mixture was incubated at room temperature for 10 min, and then the absorbance value was measured at 700 nm. The Fe^3+^ total reducing ability was calculated as follows:

(11) $${\mathrm{Fe}}^{3+}{\;\mathrm{total}\;\mathrm{reducing}\;\mathrm{ability}\;=\;A}_{1}{\;-\;A}_{0}$$

where A_1_ is the absorption value of mixture and A_0_ is the absorption value of 60% ethanol solution instead of sample solution.

### 4.5. Statistical Analysis

Analysis of variance (ANOVA) and Duncan’s multiple range tests at *p* < 0.05 were conducted to determine the differences between treatments. Statistical analyses were carried out using the Design-Expert v11 software (Stat-Ease, Inc., Minneapolis, MN, USA).

## 5. Conclusions

Through this study, a mild method for efficient extraction and purification of PNF was established. The antioxidant in vitro activity assay of flavonoids showed that P-PNF had stronger antioxidant capacity than PNF, indicating that it should be investigated as a natural antioxidant. However, this study did not elucidate the molecular structure of PNF, and there were no cell or mouse studies to assess the antioxidant properties of PNF in vivo, both of which warrant further investigation. In conclusion, the experimental results of the study provide important insights for the development and utilization of PNF while offering theoretical guidance for the further processing and application of industrial by-product resources.

## Figures and Tables

**Table 1:** Design proposal and result of response surface experiment.

Test Number	X_1_ Cellulase Dosage/(U/g)	X_2_ Material/liquid Ratio/(g/mL)	X_3_ Extraction Time/h	Flavonoid Yield/%	ABTS ^+^ Clearance Rate/%
1	75 (−1)	1:10 (−1)	2 (0)	3.07	78.10
2	105 (+1)	1:10 (−1)	2 (0)	3.12	75.18
3	75 (−1)	1:30 (+1)	2 (0)	3.19	79.71
4	105 (+1)	1:30 (+1)	2 (0)	3.28	84.08
5	75 (−1)	1:20 (0)	1 (−1)	3.30	84.67
6	105 (+1)	1:20 (0)	1 (−1)	3.27	83.36
7	75 (−1)	1:20 (0)	3 (+1)	3.28	86.86
8	105 (+1)	1:20 (0)	3 (+1)	3.24	84.53
9	90 (0)	1:10 (−1)	1 (−1)	2.74	80.15
10	90 (0)	1:30 (+1)	1 (−1)	3.21	84.23
11	90 (0)	1:10 (−1)	3 (+1)	2.64	80.88
12	90 (0)	1:30 (+1)	3 (+1)	3.17	85.84
13	90 (0)	1:20 (0)	2 (0)	3.41	87.59
14	90 (0)	1:20 (0)	2 (0)	3.38	88.32
15	90 (0)	1:20 (0)	2 (0)	3.46	87.01

**Table 2:** Analysis of variance of optimal regression equation describing flavonoid yield and ABTS^+^ clearance rate by response surface methodology.

Source	DF	Seq SS	Adj SS	*F*-Value	*p*-Value
Regression	9	0.644568	0.071619	4.80	0.049 *
Linear	3	0.209925	0.069975	4.69	0.065
Square	3	0.433318	0.144439	9.68	0.016 *
Interaction	3	0.001325	0.000442	0.03	0.992
Residual Error	5	0.074592	0.014918	14.56	0.065
Lack-of-Fit	3	0.071325	0.023775		
Pure Error	2	0.003267	0.001633		
Total	14				
Regression	9	198.433	22.0482	24.50	0.001 *
Linear	3	52.436	17.4787	19.42	0.003 *
Square	3	132.258	44.0859	48.98	0.000 *
Interaction	3	13.740	4.5799	5.09	0.056
Interaction	3	13.740	4.5799	5.09	0.056
Residual Error	5	4.500	0.9001	2.81	0.273
Lack-of-Fit	3	3.639	1.2129		
Pure Error	2	0.862	0.4309		
Total	14				

Note: Above the dotted line is the analysis of variance of flavonoids; below the dotted line is the analysis of variance of ABTS^+^ clearance rate. * Significance at *p* < 0.05.

**Table 3:** Coefficient test of optimal mode describing flavonoid yield and ABTS^+^ clearance rate by response surface methodology.

Item	Coefficient	St Dev	T	*p*-Value
Constant	3.41667	0.07052	48.451	0.000
X_1_	0.00875	0.04318	0.203	0.847
X_2_	0.16000	0.04318	3.705	0.014 *
X_3_	−0.02375	0.04318	−0.550	0.606
X_1_^2^	0.04042	0.06356	0.636	0.553
X_2_^2^	−0.29208	0.06356	−4.595	0.006 *
X_3_^2^	−0.18458	0.06356	−2.904	0.034 *
X_1_×X_2_	0.01000	0.06107	0.164	0.876
X_1_×X_3_	−0.00250	0.06107	−0.041	0.969
X_2_×X_3_	0.01500	0.06107	0.246	0.816
X_1_	−0.2737	0.3354	−0.816	0.452
X_2_	2.4438	0.3354	7.285	0.001 *
X_3_	0.7125	0.3354	2.124	0.087
X_1_^2^	−3.1463	0.4937	−6.372	0.001 *
X_2_^2^	−5.2263	0.4937	−10.585	0.000 *
X_3_^2^	0.3612	0.4937	0.732	0.497
X_1_×X_2_	1.8225	0.4744	3.842	0.012 *
X_1_×X_3_	−0.2550	0.4744	−0.538	0.614
X_2_×X_3_	0.2200	0.4744	0.464	0.662

Note: * Significance at *p* < 0.05.

**Table 4:** Results of orthogonal experiment.

Test Number	A	B	C	D	Flavonoid Recovery (%)
	Sample Concentration (mg/mL)	pH	Eluent Concentration (%)	Water Washing Volume (BV)	
1	1.0	5	30	3	43.54
2	1.0	6	50	4	64.07
3	1.0	7	70	5	66.91
4	1.5	5	50	5	76.07
5	1.5	6	70	3	64.05
6	1.5	7	30	4	37.69
7	2.0	5	70	4	75.67
8	2.0	6	30	5	32.73
9	2.0	7	50	3	71.17
K_1j_	174.52	195.28	113.95	178.76	531.9(T)
K_2j_	177.81	160.84	211.30	177.43	
K_3j_	179.57	175.76	206.63	175.71	
k_1j_	58.17	65.09	37.99	59.59	59.1
k_2j_	59.27	53.62	70.43	59.14	
k_3j_	59.86	58.59	68.88	58.57	
R_j_	1.86	11.48	32.45	1.02	

**Table 5:** Analysis of variance of orthogonal array experiments.

Source of Variation	SS	df	MS	F	F_a_	Significant
A (Sample Concentration)	4.3805	2	2.1902	2.8100	F_0.05 (2,8)_ = 4.46	
B (pH)	198.3023	2	99.1511	127.2093		**
C (Eluent Concentration)	2009.2594	2	1004.6297	1288.9232	F_0.01 (2,8)_ = 8.65	**
Error	1.5589	2	0.7794			

Note: The D factor with the smallest difference was considered as the error term. SS–sum-of-squares, df–degrees of freedom, MS–mean squares. ** Extreme significance at *p* < 0.01.

## Data Availability

Most of the recorded data are available in all Tables and Figures in the manuscript.

## Appendix A

**Table A1:** Composition of *Pinus koraiensis* nut-coated film.

Indicator	Content (%)
Protein	4.63
Axunge	0.18
Total sugar	2.79
Reducing sugar	1.90
Flavone	2.83
Polyphenol	2.96
VC	0.03

**Table A2:** Analysis of variance of optimal regression equation describing flavonoid yield and ABTS clearance rate by response surface methodology.

Source	DF	Seq SS	Adj SS	F	P
Regression	9	0.644568	0.071619	4.80	0.049
Linear	3	0.209925	0.069975	4.69	0.065
Square	3	0.433318	0.144439	9.68	0.016
Interaction	3	0.001325	0.000442	0.03	0.992
Residual Error	5	0.074592	0.014918	14.56	0.065
Lack-of-Fit	3	0.071325	0.023775		
Pure Error	2	0.003267	0.001633		
Total	14				
Regression	9	198.433	22.0482	24.50	0.001
Linear	3	52.436	17.4787	19.42	0.003
Square	3	132.258	44.0859	48.98	0.000
Interaction	3	13.740	4.5799	5.09	0.056
Interaction	3	13.740	4.5799	5.09	0.056
Residual Error	5	4.500	0.9001	2.81	0.273
Lack-of-Fit	3	3.639	1.2129		
Pure Error	2	0.862	0.4309		
Total	14				

Note: Above the dotted line is the analysis of variance of flavonoids; below the dotted line is the analysis of variance of ABTS clearance rate. SS–sum-of-squares, df–degrees of freedom, MS–mean squares.

**Table A3:** Adsorption and desorption capacity of 5 macroporous resins.

Serial Number	Resin Model	Polarity	Specific Surface Area (m^2^/g)	Balanced Concentration of Flavonoids in Solution (mg/mL)	Adsorption Amount (mg/g)	Adsorption Rate (%)	Eluent Mass Concentration (mg/mL)	Desorption Rate (%)
1	AB-8	Weak polarity	450–500	0.06557	12.9957	95.1965	0.7958	61.2385
2	HP-20	Non-level	550–600	0.10134	12.6381	92.5764	0.7661	60.6132
3	HPD600	Polarity	550–600	0.08942	12.7572	93.4498	0.8376	65.6542
4	HPD826	Hydrogen bond	500–600	0.12519	12.3996	90.8297	0.6528	52.6442
5	D101	Group polarity	480–520	0.11327	12.5188	91.7031	0.7279	58.1429
